# Validation and deployment of a direct saliva real-time RT-PCR test on pooled samples for COVID-19 surveillance testing

**DOI:** 10.1371/journal.pone.0261956

**Published:** 2021-12-30

**Authors:** Amanda Rainey, Austin Pierce, Xiaoyun Deng, Luis A. Actis, Philip Smith, Andor J. Kiss, Timothy J. Wilson

**Affiliations:** 1 Department of Microbiology, Miami University, Oxford, Ohio, United States of America; 2 Department of Kinesiology, Nutrition, and Health, Miami University, Oxford, Ohio, United States of America; 3 Center for Bioinformatics and Functional Genomics, Miami University, Oxford, Ohio, United States of America; University of Helsinki: Helsingin Yliopisto, FINLAND

## Abstract

A direct, real-time reverse transcriptase PCR test on pooled saliva was validated in 2,786 participants against oropharyngeal swabs. Among asymptomatic/pre-symptomatic participants, the test was found to be in 99.21% agreement and 45% more sensitive than contemporaneous oropharyngeal swabs. The test was then used for surveillance testing on 44,242 saliva samples from asymptomatic participants. Those whose saliva showed evidence of SARS-CoV-2 within 50 cycles of amplification were referred for confirmatory testing, with 87% of those tested by nasal swab within 72 hours receiving a positive diagnostic result on Abbott ID NOW or real-time PCR platforms. Median Ct values on the saliva PCR for those with a positive and negative confirmatory tests was 30.67 and 35.92 respectively, however, binary logistic regression analysis of the saliva Ct values indicates that Ct thresholds as high as 47 may be useful in a surveillance setting. Overall, data indicate that direct RT-PCR testing of pooled saliva samples is an effective method of SARS-CoV-2 surveillance.

## Introduction

SARS-CoV-2 is a ssRNA virus of the family Coronaviridae and the etiologic agent responsible for the disease, COVID-19 [1–3]. The likely transmission of SARS-CoV-2 from bats to humans represents the third documented outbreak of an animal coronavirus in humans within the last two decades [1] and is an ongoing public health concern [2–4]. The advent of the SARS-CoV-2 pandemic led to a sudden increase in demand for diagnostic testing resources. In addition to testing for patients with symptoms of COVID-19, additional high-volume testing operations were initiated for public health efforts by government agencies, employers, and educational institutions. Along with this increased demand for testing came challenges surrounding the cost, capacity, supply availability, and time-to-results for testing-based public health programs. Within public health surveillance and screening programs, a rapid turnaround time is essential to identifying and containing outbreaks [4, 5], and so solutions for obtaining rapid and reliable results were necessary within large-scale COVID-19 testing programs.

Saliva-based testing for SARS-CoV had been previously documented [6, 7], and saliva testing showed promise for identifying cases of SARS-CoV-2 [8, 9], making it an attractive option for non-invasive testing of asymptomatic individuals. Additional testing efficiencies were demonstrated through RNA sample pooling and amplification of SARS-CoV-2 genes from saliva without RNA extraction [10–12], however, no studies had attempted to combine the processes on a large scale, despite indications that such a program could be beneficial [10]. In order to create a cost-effective surveillance testing program that did not compete with diagnostic testing resources, an in-house surveillance testing program was developed, validated, and deployed. This report is a retrospective analysis of the performance of a pooled direct saliva test used for SARS-CoV-2 surveillance on a university campus and provides methodological guidance to inform best practices.

## Materials and methods

### Ethics statement

All participants in the surveillance testing program were Miami University undergraduate and graduate students attending on-campus classes and activities and living on campus or off-campus in Oxford, OH. Those participating the saliva testing program provided informed consent for data sharing among authorized individuals employed by the University and Student Health systems. Those declining consent for data sharing still participated in surveillance testing through existing diagnostic testing routes, but are not included in this study. The requirement for additional informed consent for retrospective analysis of anonymized data was waived after review by the Miami University Institutional Review Board (protocol reference number: 03863e). The fully anonymized minimal dataset underlying this analysis can be obtained from the Miami University Scholarly Commons: https://sc.lib.miamioh.edu/handle/2374.MIA/6767.

### Testing period and participant demographics

The validation phase of the testing platform was carried out in November 2020, and the saliva surveillance testing program was testing students from January through May 2021. Demographic information such as age, sex, race, and ethnicity were not collected for this retrospective analysis. The sample pool was made up entirely of undergraduate and graduate students from the Miami University–Oxford campus, and so the sample can be considered to be representative of a medium-sized, public university in the United States.

### Sampling of participants

Approximately 800 students were sampled per day, with replacement after one week. We utilized a quota-based adaptive cluster sampling approach, with quotas for campus strata calculated based on campus COVID prevalence accounted for by each strata. Strata included on campus Greek Life-affiliated students, on campus freshman non-Greek Life students, on campus non-freshman non-Greek Life students, off campus Greek Life undergraduate students, off campus non-Greek Life undergraduate students, off campus graduate students, and students who had tested positive for COVID-19 > 90 days. Each sampling day, the percent of community COVID-19 prevalence accounted for by each group was estimated using testing positivity rates and strata size, and a sampling quota was calculated for that strata based on this percentage. For example, if we estimated that a strata accounted for 30% of community COVID prevalence, that strata’s quota would be calculated as .30 * 800. Within strata, sampling was weighted based on the size of the student’s congregate living environment, with students living with or around more students having a greater sampling weight. Our adaptive cluster sampling approach was based on congregate living. When a positive case was identified within a congregate living area, all others living in congregation with the person who tested positive were invited for a test. N = 9,825 students were eligible to be tested at the outset of the spring semester. Those with a previous positive COVID-19 diagnostic test within the previous 90 days were excluded from the pool as were students currently experiencing symptoms of COVID-19.

### Saliva collection for surveillance testing

A volume of 100μL of 178 mM Tris-borate/4 mM EDTA (2x-TBE, Sigma-Aldrich, Saint Louis, MO) was added to 5-mL conical screw cap tubes (Eppendorf, Enfield, CT or MTC Bio, Metuchen, NJ). Samples collection was supervised by clinical staff at the Miami University Student Health Center. Participants were asked to provide a saliva sample into the collection tube matching the volume of the TBE buffer present. After collection, saliva samples were inactivated by incubation in a convection oven at 95°C for 30 minutes and stored at room temperature overnight until analysis. During the deployment phase, samples collected on Saturdays were frozen at -20°C until analysis the following Monday.

### Pooling and RT-PCR testing of saliva samples

For sample pooling, 60 μL 1xTBE with 1% TWEEN-20 (TBE-T) was first added to each well of a 96-well skirted PCR plate (PurePlus 0.1 mL 96-well, Thomas Scientific, Swedesboro, NJ). Fifteen microliters of inactivated saliva per sample were added to the pooling plates. Pool size was determined by the number of samples received and the capacity of the 384-well reaction plates. Pools of 2 were run up to a capacity of 764 samples, above which, pools of 3 were used. Samples run individually, including retests of positive, failed, or inconclusive pools, were diluted with TBE-T at a 2:1 buffer to saliva ratio in the pooling plates. After sample pooling, plates were sealed and mixed by vortexing for 60 seconds at 1650 rpm using an Eppendorf MixMate. Sample plates were then centrifuged at 3,000 rpm for 5 seconds on a Sorvall Legend X1R to move samples to the bottom of the wells.

For reverse-transcriptase polymerase chain reactions (RT-PCR), the protocol was adapted from Ranoa et al. [11]. A master mix was prepared so that each reaction contained 5 μL TaqPath 1-Step RT-PCR reaction mix (ThermoFisher), 3 μL water, 1 μL TaqPath COVID-19 Combo Kit probe mix (ThermoFisher), and 1 μL MS2 phage control. For each well in the 384-well reaction plate, 15 μL of master mix and 5 μL of pooled saliva sample were dispensed to each well using an Eppendorf epMotion 5073m automated liquid handling system. The master mix reservoir was kept on wet ice and the reaction plate was maintained on the TMX chiller at 4°C throughout the pipetting procedure. Positive (vendor supplied SARS-CoV-2 RNA) and negative (TBS-T) reaction controls were added to each plate manually. At the completion of the plate setup, the reaction plate was sealed and vortexed at 2,600 rpm for 30 seconds using the MixMate vortexer. The plate was again centrifuged at 3,000 RPM for 5 seconds before loading into the Quantstudio 7-Flex real-time PCR instrument (ThermoFisher). Cycling conditions were as follows: 25°C for 2 minutes; 53°C for 10 minutes; 95°C for 2 minutes; followed by 50 cycles of amplification at 95°C for 3 seconds (ramp rate 1.9°C/s) then 60°C for 30 seconds (1.6°C/s ramp rate).

### Workflow, results, and referral criteria during deployment phase

Sample collection and PCR analyses were performed Monday–Saturday each week on samples acquired the collection day. Pooled testing was used Monday–Friday (84 testing days), and samples were run without pooling on Saturdays (16 days) to facilitate early aggregate data reporting and timely retest referrals. Pooled samples, 2–3 per reaction, were diluted and pooled before transferring to the 384 well reaction plate. After the initial real-time PCR, samples which were part of positive, inconclusive, and failed pools were retested individually. Positive pools or samples were defined as those showing clear evidence of exponential amplification of both ‘*N*’ and ‘*ORF1ab*’ genes within 50 cycles; inconclusive showed amplification of only 1 SARS-CoV-2 marker, negative showed no amplification of SARS-CoV-2 markers, but successful amplification of MS2; and failed reactions were those that did not clearly amplify MS2. In total, 44,242 samples were processed during the deployment phase, with 39,714 run first as pooled samples and 4,528 run individually. A total of 19,806 initial PCR reactions were run as pools of 2, and 34 reactions were run as pools of 3. After the individual sample retest, participants whose samples showed positive or inconclusive evidence of SARS-CoV-2 were referred for diagnostic testing. No Ct threshold was used for the purposes of referral for diagnostic testing. Participants whose samples did not show evidence of SARS-CoV-2 were not referred for diagnostic testing, regardless of MS2 amplification status.

### Diagnostic testing

During the validation phase, oropharyngeal (OP) swabs were collected from asymptomatic participants in the surveillance testing pool by clinical staff and sent to external diagnostic laboratories for RT-PCR testing. Saliva samples were contemporaneously collected from volunteers among these participants and processed as indicated below. Volunteers who tested negative on their initial OP swab, but whose saliva sample showed evidence of SARS-CoV-2 RNA, were referred for retesting by Abbott ID NOW rapid testing. Retesting by OP swab on the Abbott ID NOW platform was performed 1–2 days after the initial saliva sample collection.

Once the saliva surveillance testing program was deployed, diagnostic testing for participants was performed following referral by the saliva surveillance program. Participants were first tested using the Abbott ID NOW system on nasal swabs collected by clinical personnel. Positive results were considered definitive. If the Abbott ID NOW system returned a negative or invalid result, the nasal swab was repeated and sent to an external diagnostic laboratory for RT-PCR testing. Confirmatory testing was performed within 1–3 days after initial saliva collection, with most confirmatory diagnostic tests taking place 2 days after saliva sample collection.

### Data analysis

For saliva PCR results, data were retrospectively analyzed using the Applied Biosystems QuantStudio Real-Time PCR Software v1.7.1. For each sample and probe, Ct value was assigned at a ΔRn value of 50,000 with auto-baseline. Statistical analysis of median Ct values was performed using Graphpad Prism versions 6 and 9. Logistic regression analysis was performed using Stata v.14 and plotted using Graphpad Prism 9.

## Results

### Validation phase

To validate pooled saliva testing, the protocol previously described by Ranoa et al. was adapted for use with pooled samples [11]. While adapting the protocol, a few observations were made: 1) Some saliva samples are highly viscous, making accurate pipetting difficult and potentially impacting reaction rates within the PCR wells; 2) some samples contain an unidentified PCR inhibitor that can block amplification of SARS-CoV-2 genes as well as the MS2 phage control RNA; 3) samples containing this PCR inhibitor can block amplification of SARS-CoV-2 genes derived from other samples in the pool, thereby causing a failed test for all samples in the pool. To solve these technical problems, and taking into account previously published estimates for prevalence-based optimal pool size [13], a pooling protocol was established that included the following adaptations: sample pool size was limited to 3 samples; samples were diluted 1:1 in TBS-T while pooling; samples in positive, inconclusive, and failed pools should all be retested individually to maximize detection and accuracy.

**Fig 1:**
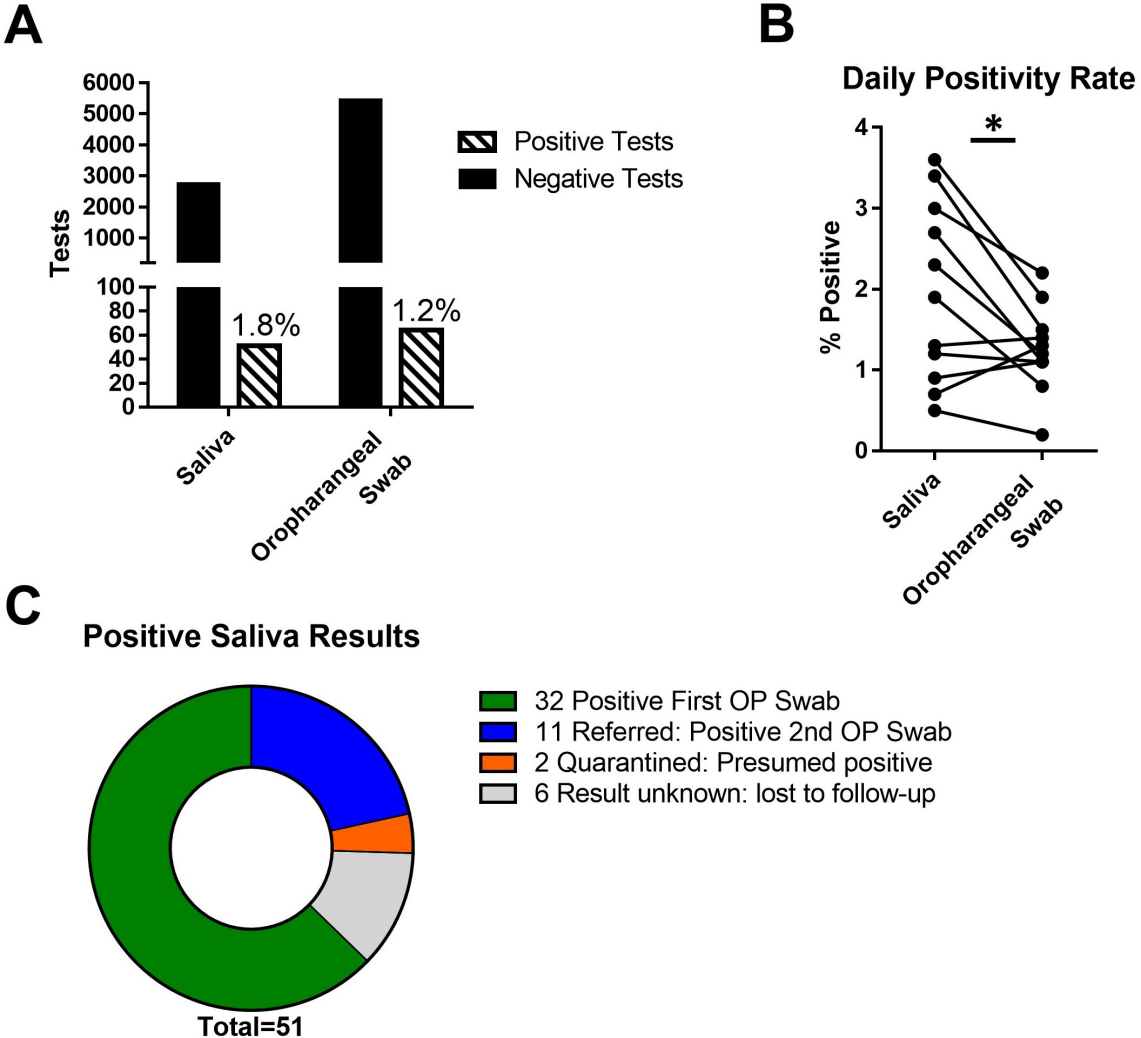
Validation of direct RT-PCR on pooled saliva for COVID-19 surveillance. A) RT-PCR positivity rates of diagnostic oropharyngeal swabs from asymptomatic participants in surveillance testing compared with pooled saliva samples collected at the same time from volunteers within the participant pool. B) Pairwise analysis of daily positivity rates between pooled saliva and oropharyngeal swab. *p<0.05. C) Summary of saliva positive results at initial sample acquisition or post-referral retest of saliva positive/swab negative participants.

With these guidelines in place, a cohort of saliva samples was obtained from volunteers in the COVID-19 surveillance testing program. Over a period of 13 days, 5510 oropharyngeal swabs were obtained from asymptomatic participants in the surveillance pool and sent for diagnostic RT-PCR testing through a contract laboratory. From among these participants, 2786 volunteers provided saliva samples for comparison. Results of these studies are summarized in Table 1 and Fig 1. Overall, saliva testing showed a significantly higher positivity rate (1.8%) compared with oropharyngeal swabs taken at the same time (1.2%, p<0.05). Examining the concordance of the test formats: of the 51 samples testing positive SARS-CoV-2 in saliva, 32 (63%) had tested positive on their contemporaneous OP swab. The remaining 19 SARS-CoV-2 saliva-positive participants were contacted to refer them for a second diagnostic test. Of these, 11 retested positive by OP swab on the Abbott ID NOW rapid test platform; 2 were already in County-ordered quarantine when contacted and were presumed positive; and 6 were lost to follow-up, either as a result of not returning for re-testing or being retested outside our testing program (Fig 1). Of the 2786 participants who donated saliva samples, 3 tested positive on their contemporary OP swab, but were negative on their saliva test. The reason for this discordance is not known, however, all three of these saliva samples were collected on a Friday or Saturday and held at room temperature until analysis the following Monday. It’s possible that the SARS-CoV-2 RNA in these samples degraded during this time, leading to a negative result. For this reason, samples to be held longer than overnight were stored at -20°C during the deployment phase.

**Table 1:** Pooled saliva testing validation data.

	**Positive**	**Negative**	**Total**	**Rate**
*Saliva*	51	2735	2786	1.8%
*OP Swab*	64	5446	5510	1.2%
	**OP Swab (+)**	**OP Swab (-)**	**Agreement**	**Kappa (95% CI)**
*Saliva (+)*	32	19	99.21%	0.740 (0.636–0.845)
*Saliva (-)*	3	2732		

Summary statistics and concordance data for observed positivity rates during the validation phase. Neutral agreement (kappa) statistic excludes retest results following referral from earlier saliva positive.

### Deployment of pooled saliva testing for COVID-19 surveillance

Following validation phase, direct real-time RT-PCR testing of pooled saliva samples for COVID-19 was deployed for surveillance testing on campus. Testing was performed Monday–Saturday each week, with the bulk of the samples processed as pooled tests except those samples processed on Saturdays, which were tested individually. Summary statistics are shown in Table 2. In total, 44,242 samples were processed with 374 samples testing positive, defined as clear amplification of “*N*” and “*ORF1ab*” genes within 50 cycles. Since the B.1.1.7 (Alpha) variant was emergent during this period and resulted in loss of “*S*” signal in the PCR results, ‘*S*’ positivity was not factored into designation of positive or negative results. Sixteen samples showed clear amplification of only one SARS-CoV-2 marker, and were designated as inconclusive. All participants with positive results, along with some inconclusive results, were given referrals for follow-up diagnostic tests. Also notable are the 226 samples for which no genes amplified, including the MS2 phage internal positive control. These were designated as failed reactions and were not referred for further testing (Table 2A). Since not all samples were processed in pools, it was possible to compare the positivity rates among samples processed as pooled vs unpooled reactions. There was no significant difference in percent positivity between pooled (95% CI 0.67–0.98) and unpooled (95% CI 0.43–1.23) samples, indicating no loss in sensitivity when using pooled reactions (Table 2B).

**Table 2:** Outcomes of surveillance testing using pooled saliva. **A)** Aggregate results from RT-PCR assays for all saliva samples received. **B)** Comparison of positivity rates for samples analyzed as pooled or individual samples. **C)** Results of diagnostic follow-up tests for participants referred due to positive detection of SARS-CoV-2 in saliva.

**A) Results Summary**	**Number**	**Percent**	
*Total samples*	44,242		
*Positive*	374	0.85%	
*Not detected*	43,626	98.61%	
*Inconclusive*	16	0.04%	
*Failed*	226	0.51%	
**B) Sample Format**	**Number**	**Positivity: % (SEM)**	
*Pooled*	39,714	0.82% (0.08)	
*Unpooled*	4,528	0.83% (0.19)	
**C) Referral Result**	**Number**	**% of Total**	**Retained**
*Total*	374		353
*Positive*	308	82.4%	87.3%
*Negative*	45	12.0%	12.7%
*Lost to follow-up*	21	5.6%	

Participants who were referred for diagnostic testing from the saliva surveillance program were initially tested on the Abbott ID NOW platform using a nasal swab collected by clinical personnel. Out of concern for reports of lower sensitivity of the ID NOW platform [14–16], participants testing negative were retested by RT-PCR using an external laboratory. Of the 374 samples referred with clear evidence of SARS-CoV-2 positivity in saliva, 308 (82%) tested positive in follow-up diagnostic tests occurring within three days of the initial saliva sample. Of these 308 positive diagnoses, 291 (94.4%) tested positive on the Abbott ID NOW test. Six participants (1.8%) tested negative by Abbott ID NOW, but positive by a contemporary RT-PCR, indicating that the Abbott ID NOW rapid test platform provides comparable sensitivity to PCR for confirmation tests using nasal swabs. Eleven participants (3.6%) tested positive through alternative clinical routes between providing a saliva sample for surveillance and receiving a diagnostic referral. Of the 374 participants referred for follow-up testing from positive saliva tests, 45 (12%) had negative nasal swabs on Abbott ID NOW and RT-PCR diagnostic platforms, and 21 (5.6%) were lost to follow-up (Table 2C). Of the participants referred for follow-up testing with an inconclusive saliva result, none tested positive on their diagnostic swab. Overall, these data indicate that direct testing of pooled saliva samples provide a sensitive and reliable method of COVID-19 surveillance.

**Fig 2:**
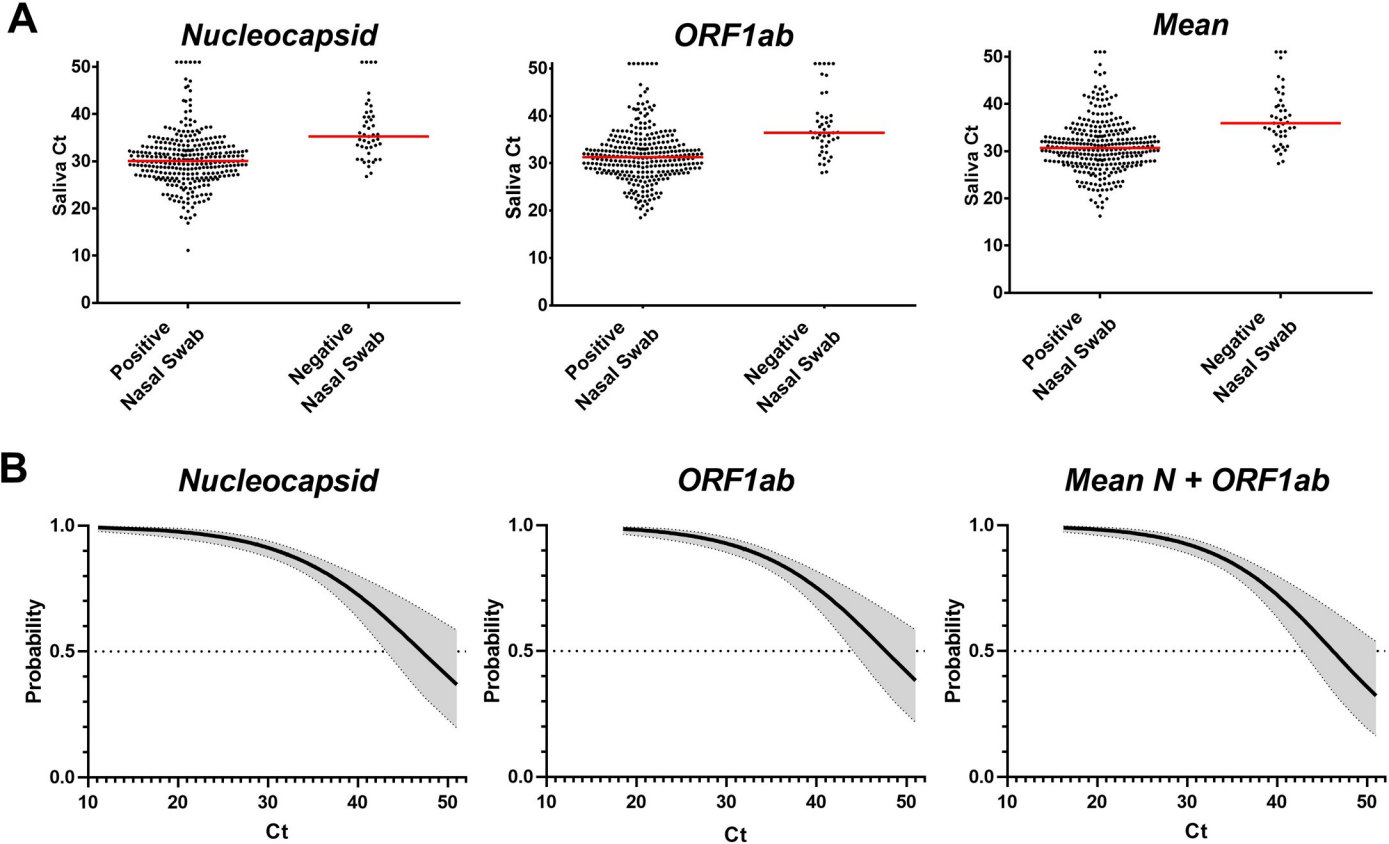
Association between saliva test Ct value and subsequent diagnosis of COVID-19. A) Ct values for two SARS-CoV-2 genes, ‘*N*’ and ‘*ORF1ab*,’ as well as their mean Ct and subsequent diagnostic result following re-test referral. Median saliva Ct values were significantly lower (p<0.0001) for those testing positive by nasal swab. Each dot represents 1 participant sample. Genes which amplified within 50 cycles, but did not cross the threshold, were assigned a Ct value of 51 for post-hoc analysis. B) Probability of a subsequent positive diagnosis based on saliva Ct value was calculated using a logistic regression model. 95% confidence intervals are shown in gray on each side of the best fit line.

Excluding those lost to follow-up, 87% (308/353) of participants who had a positive saliva test received a positive diagnosis for COVID-19. The 13% discordance likely reflects the increased sensitivity of testing for SARS-CoV-2 in saliva when compared with the nasal swab [8, 9, 17, 18], but it may also be an indicator of the infection kinetics, whereby individuals at late stages of the infection course may test negative soon after a positive saliva test. To improve the utility of highly sensitive RT-qPCR tests in clinical and public health efforts, it has been proposed that the PCR cycle threshold (Ct) value should be taken into consideration when reporting results [19, 20]. If RNA is purified from clinical samples, it becomes possible to quantitate patient viral copy number based on the Ct value for the target genes [21]. However, this method is complicated by a lack of an effective and universally appropriate method for standardization of the testing process from sample collection through analysis of qPCR results. In addition, there is great uncertainty in how to infer infectivity from qPCR tests [22, 23]. When amplifying SARS-CoV-2 directly from saliva, PCR efficiency varies widely from one sample to the next, making absolute quantitation impractical, if not impossible. This was evident from the Ct values of the MS2 phage control (included with the ThermoFisher TaqPath COVID-19 Combo kit), which was included at the same concentration in every sample. Amplification of MS2 gave a median Ct among SARS-CoV-2 positive samples of 30.8, with a range of 27.3 to >50 and an interquartile range (IQR) of 29.7–32.8. Among the entire pool of samples, MS2 failed to amplify in 0.5% of cases, even upon individual retesting. Therefore, inferring viral copy number or infectivity from the Ct value of a direct saliva test is very difficult, and may not be practical. Despite this intrinsic variability, the potential of the Ct values of the SARS-CoV-2 amplicons to predict subsequent diagnosis was examined. Individual *N* or *ORF1ab* Ct values, or the mean Ct value from saliva positive participants was compared to their follow-up diagnostic swab result. The *S* gene was not included in this analysis owing to its failure to amplify from B.1.1.7-infected individuals [24]. Participants with a positive COVID-19 diagnosis from their post-surveillance nasal swab had lower median Ct values (Mann-Whitney p<0.0001) on their saliva surveillance test than those with a negative diagnostic swab, with a median Ct of 30.67 (IQR: 27.57–33.59) for positives and 35.92 (IQR: 33.16–40.22) for negatives (Fig 2A). Despite these differences in median Ct values, there was not a clear demarcation in saliva Ct values between those that went on to test positive or negative by nasal swab. To determine at what value, if any, a Ct threshold may be warranted, a binary logistic regression model was applied to determine if the Ct value of the saliva test could be used to predict diagnostic positivity. From this analysis, it was found that a qualitatively positive result by saliva RT-PCR was more likely than not to produce a positive diagnostic nasal swab out to a Ct value of 47 (Fig 2B), indicating that a strict Ct cutoff for surveillance positivity earlier than cycle 47 would have reduced the number of cases found and potentially adversely impacted the effectiveness of the surveillance testing program.

## Discussion

In summary, we are reporting the validation and successful deployment of a direct saliva testing method using pooled saliva samples for SARS-CoV-2 surveillance on a university campus. In validation studies, direct RT-PCR testing of pooled saliva was found to be more sensitive than diagnostic testing of oropharyngeal swabs, and 100% of saliva positive participants for whom diagnostic results were available showed a positive oropharyngeal swab either at the same time or within 3 days of saliva sample acquisition. This indicated that the direct RT-PCR testing of pooled saliva samples was a viable option for surveillance testing for which the risk of false negatives is relatively low in comparison to the overall increase in sensitivity while maintaining excellent specificity.

While real-time PCR, commonly referred to as quantitative PCR (qPCR), is a technique that lends itself to quantitative analysis of template concentrations, diagnostic results from qPCR assays are commonly reported as a binary result of “positive” for samples showing amplification before a specified Ct cutoff or “not detected” for samples showing either no target amplification or amplification after the specified Ct cutoff. As a result of this binary reporting approach, the appropriate Ct cutoff for use in SARS-CoV-2 surveillance has been a matter of some controversy [19, 20, 22, 23]. Since all participants in the testing program were asymptomatic at the time of testing, it is not possible to distinguish those at the earliest phases of infection from those nearing recovery. Furthermore, when directly testing biological samples, without prior RNA extraction, PCR efficiency is highly variable. For these reasons, specific Ct cutoffs for qualitative determination of positive or negative results should be carefully evaluated prior to use in a surveillance setting, and we recommend against attempting to infer infectivity from a direct saliva PCR assay.

Retrospective comparison of pooled and unpooled samples during the deployment phase found that the use of sample pooling for up to three samples per pool did not significantly decrease test sensitivity, while realizing significant cost gains over using diagnostic testing for surveillance. It is notable that pooling of samples is slightly more labor-intensive than individual sample testing, and so cost-savings are found principally in reagents and consumables use. In this case, the average reagent cost per sample tested was just over $10 when using pooled testing, or approximately half the cost of testing all samples individually. Overall, this amounts to a small fraction of the cost of contract diagnostic saliva testing services. Further significant cost savings could likely be achieved through validation of multiplexed, non-proprietary CDC or WHO primer and probe sets. In addition, optimizing the size of sample pools based on observed prevalence and reaction failure rates could further increase efficiency. This study is a retrospective analysis of results from deployment of a functioning COVID-19 surveillance operation, and so the absolute limits of sample pooling were not explored. Nevertheless, these results establish the benefits of combining direct RT-PCR testing of saliva together with sample pooling to achieve cost savings in an environment of finite resources, supply-chain challenges, or limited access to high-throughput sample preparation instrumentation. This strategy may be especially valuable as a public health surveillance tool to complement diagnostic testing for the management of SARS-CoV-2, as well as other respiratory illnesses, within semi-closed communities [4].
